# Supplementary material for: An ultralight, tiny, flexible six-axis force/torque sensor enables dexterous fingertip manipulations
Source: Nat Commun. 2025 Jul 1;16:5693. doi: 10.1038/s41467-025-60861-8 (PMC12214963; doi:10.1038/s41467-025-60861-8)
Supplement: Supplementary file 1 — Supplementary Information [file 41467_2025_60861_MOESM1_ESM.pdf]

Supplementary Information for

An ultralight, tiny, flexible six-axis force/torque sensor enables dexterous fingertip manipulations

Qian Mao[1], Zijian Liao[1], Shiqiang Liu[1], Jinfeng Yuan[1] and Rong Zhu[1]*
Corresponding author: Email: zr_gloria@mail.tsinghua.edu.cn

The PDF file includes:

Supplementary Notes
Figs. S1 to S14
Table. S1

Other Supplementary Information for this manuscript include the following:

Supplementary Movies 1 to 3

Supplementary Notes

Temperature compensation and bending compensation of the sensor

For the sake of simplicity, we use unit 1 as an example to explain the temperature compensation of the sensor here (shown in the Figure S3).

Due to the high resistance of the cold-film (~ 300 Ω), its heat power can be ignored. Therefore, its temperature is nearly the same as the ambient temperature, and its resistance can be expressed as:

$$R_{c1} = R_{c10}(1 + \alpha_c T) \tag{1}$$

where R_{c10} is the resistance of the cold-film at 0 °C, α_c is its temperature coefficient resistance (TCR), and T represents the ambient temperature. According to the principle of voltage division in circuits:

$$\frac{U_{t1} - U_{c1}}{U_{c1}} = \frac{R_{b1}}{R_{t1} + R_{c1}} \tag{2}$$

$U_{t1}, U_{c1}, R_{b1}, R_{t1}$ refers to the corresponding symbols in Figure S3. Combining equation (1) and equation (2), it can be obtained that the ambient temperature T satisfies:

$$\frac{U_{c1}}{U_{t1} - U_{c1}} = \frac{R_{c10} \cdot \alpha_c}{R_{b1}} \cdot T + \frac{R_{t1} + R_{c10}}{R_{b1}} \tag{3}$$

Here, we define:

$$\begin{cases} \beta = \dfrac{U_{c1}}{U_{t1} - U_{c1}} \\ A = \dfrac{R_{c10} \cdot \alpha_c}{R_{b1}} \\ B = \dfrac{R_{t1} + R_{c10}}{R_{b1}} \end{cases} \tag{4}$$

Then equation (3) can be simplified as:

$$\beta = A \cdot T + B \tag{5}$$

where A and B are constants.

When the Wheatstone bridge is balanced, their resistances satisfiy the following relationship:

$$\frac{R_{a1}}{R_{b1}} = \frac{R_{h1}}{R_{t1} + R_{c1}} \tag{6}$$

R_{a1} refers to the corresponding symbols in Figure S3. The temperature difference between the hot film and the ambient temperature is denoted as ΔT. Therefore, we can obtain:

$$R_{h1} = R_{h10}\big(1 + \alpha_h(T + \Delta T)\big) \tag{7}$$

Then equation (6) can be expressed as:

$$\frac{R_{a1}}{R_{b1}} = \frac{R_{h10}\big(1 + \alpha_h(T + \Delta T)\big)}{R_{t1} + R_{c10}(1 + \alpha_c T)} \tag{8}$$

where R_{h10} is the resistance of the hot-film at 0 °C, α_h is its TCR. Since α_h and α_c are almost the same, they are collectively recorded as α. In this circuit, we set:

$$\frac{R_{a1}}{R_{b1}} = \frac{R_{h10}}{R_{c10}} \tag{9}$$

Combining equations (8) and (9), we can get:

$$\Delta T = \frac{R_{t1}}{\alpha \cdot R_{c10}} \tag{10}$$

This shows that the temperature difference ΔT between the hot-film and the environment is constant and not affected by the ambient temperature. Therefore, temperature compensation is realized by this circuit.

Further, considering the sensor is working under bending states, the equation (6) can be expressed as:

$$\frac{R_{a1}}{R_{b1}} = \frac{R_{h1}}{R_{t1} + R_{c1}} = \frac{R_{h10}\big(1 + \alpha_h(T + \Delta T)\big)(1 + K_h\varepsilon_h)}{R_{t1} + R_{c10}(1 + \alpha_c T)(1 + K_c\varepsilon_c)} = \frac{R_{h10}(1 + \alpha_h(T + \Delta T) + K_h\varepsilon_h + \alpha_h(T + \Delta T)K_h\varepsilon_h)}{R_{t1} + R_{c10}(1 + \alpha_c T + K_c\varepsilon_c + \alpha_c T K_c\varepsilon_c)} \tag{11}$$

where ε_h and ε_c are the strains of the hot-film and cold-film, K_h and K_c are the gauge factors of the hot-film and cold-film. Since the hot-film and cold-film have similar geometric shapes and the fabrication process is exactly the same, it can be considered that $K_h\varepsilon_h = K_c\varepsilon_c$. Furthermore, ignoring high-order small quantities $\alpha_h(T + \Delta T)K_h\varepsilon_h$ and $\alpha_c T K_c\varepsilon_c$, we can get:

$$\Delta T = \frac{R_{t1}}{\alpha \cdot R_{c10}} - \frac{K_h\varepsilon_h - K_c\varepsilon_c}{\alpha} = \frac{R_{t1}}{\alpha \cdot R_{c10}} \tag{12}$$

which indicates that bending states will not affect the constant temperature difference ΔT, i.e. achieves bending compensation.

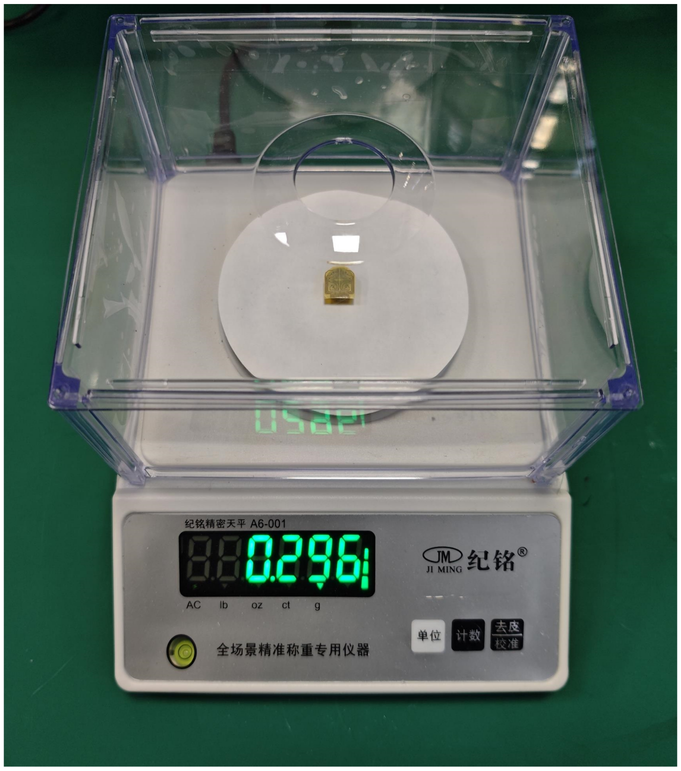

Fig. S1. The tiny six-axis force/torque sensor weighs less than 0.30 g.

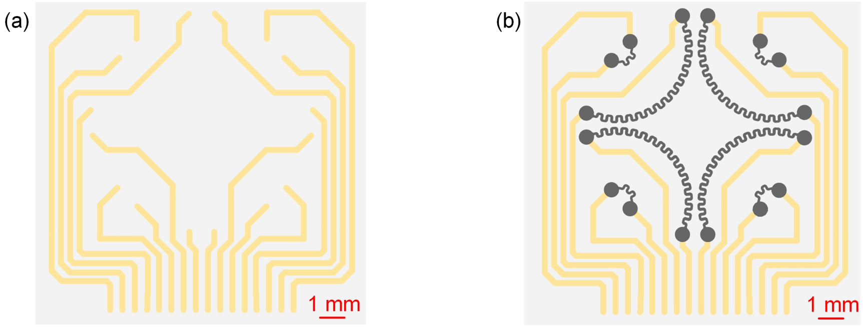

Fig. S2. Circuit routing of the FPCB. (a) Wire arrangement on the FPCB. (b) Sensors arrangement on the FPCB.

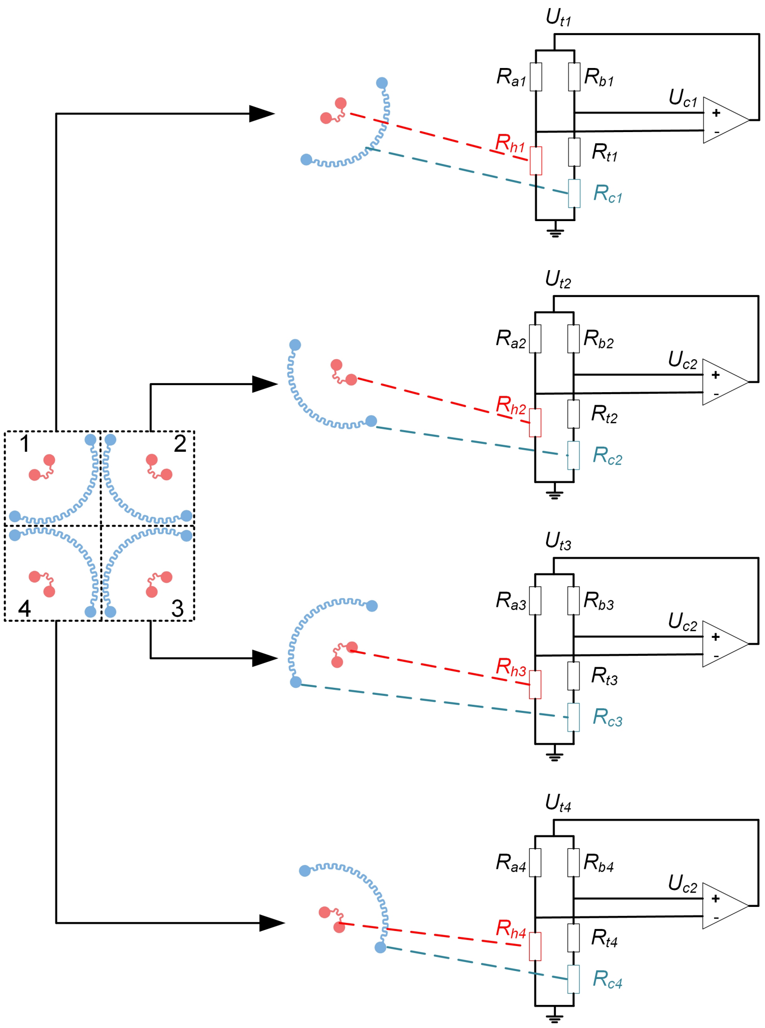

Fig. S3. The schematic diagram of the constant temperature difference (CTD) circuit.

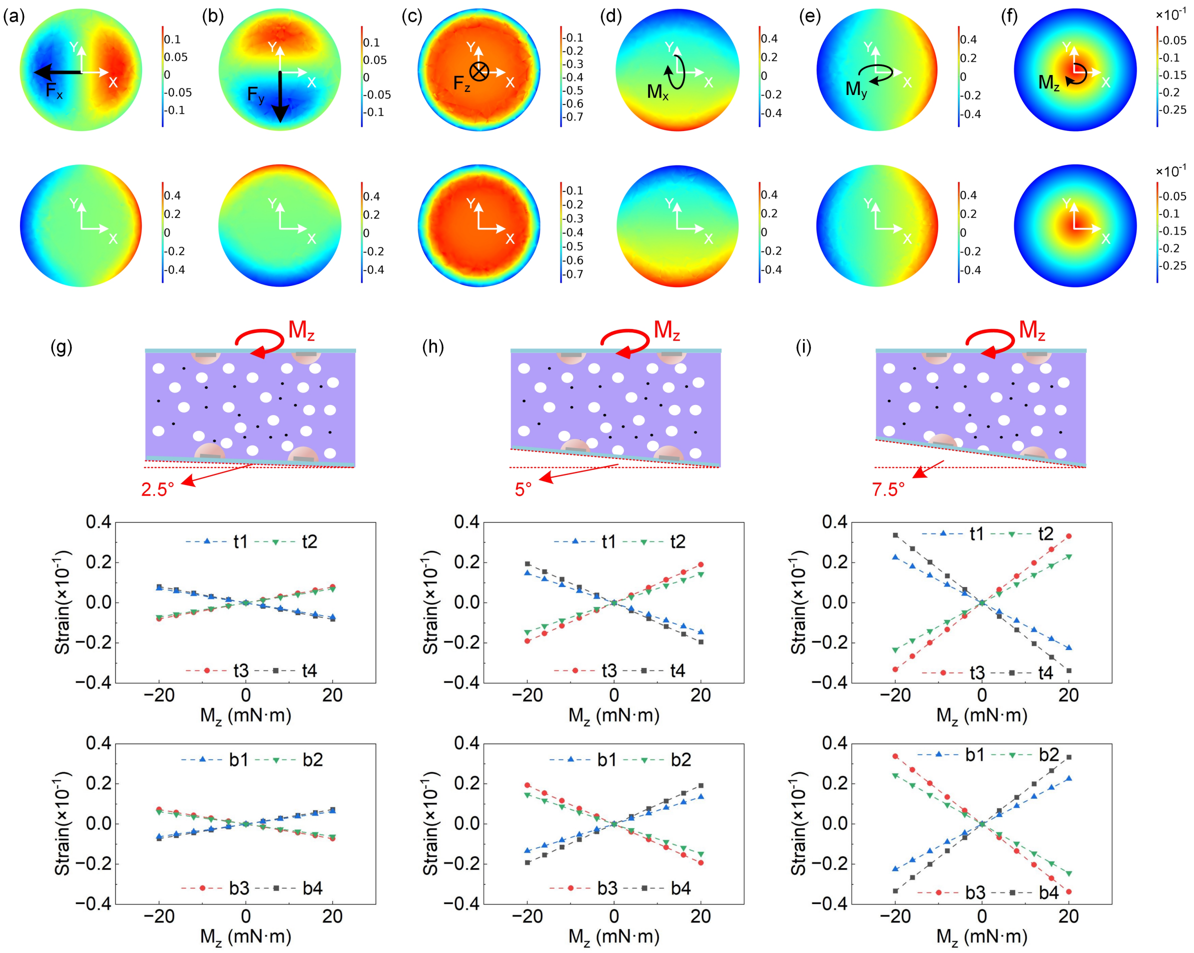

Fig. S4. Parametric optimization of 5° bevel between the top and bottom sensing layers. (a)-(f) Top: the strain cloud diagram of the A-A plane when there is no bevel (0°). Bottom: the strain cloud diagram of the B-B plane when there is no bevel (0°). The results show that both F_z and M_z cause a similar circular strain pattern, as shown in (c) and (f). **Therefore, we tailor a slight bevel to differentiate F_z and M_z.** (g)-(i) Top: schematic diagrams of the sensor with different bevels (2.5°, 5°, and 7.5°). Middle: the simulation results of the relationship between the strains at the sensing units on the top layers and applied torque M_z. Bottom: the simulation results of the relationship between the strains at the sensing units on the bottom layers and applied torque M_z. As the bevel increases from 2.5° to 7.5°, the strain under the same torque also increases, which is beneficial to the measurement of torque. However, a large bevel angle is not conducive to the practical applications of the sensor, so here we choose a bevel angle of 5°.

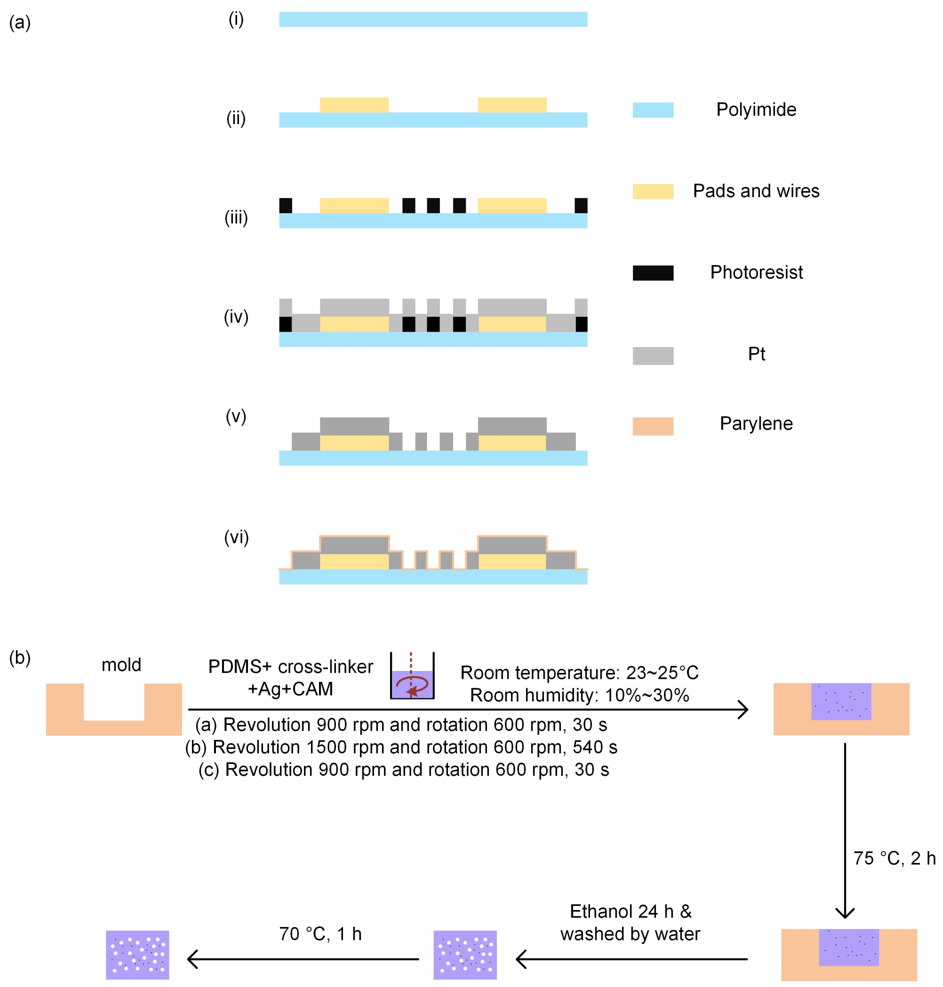

Fig. S5. The schematic diagram of the fabrication process. (a) Fabrication processes of the top sensing layer and the bottom sensing layer. (b) Fabrication process of the porous material.

(a)

(b)

(c)

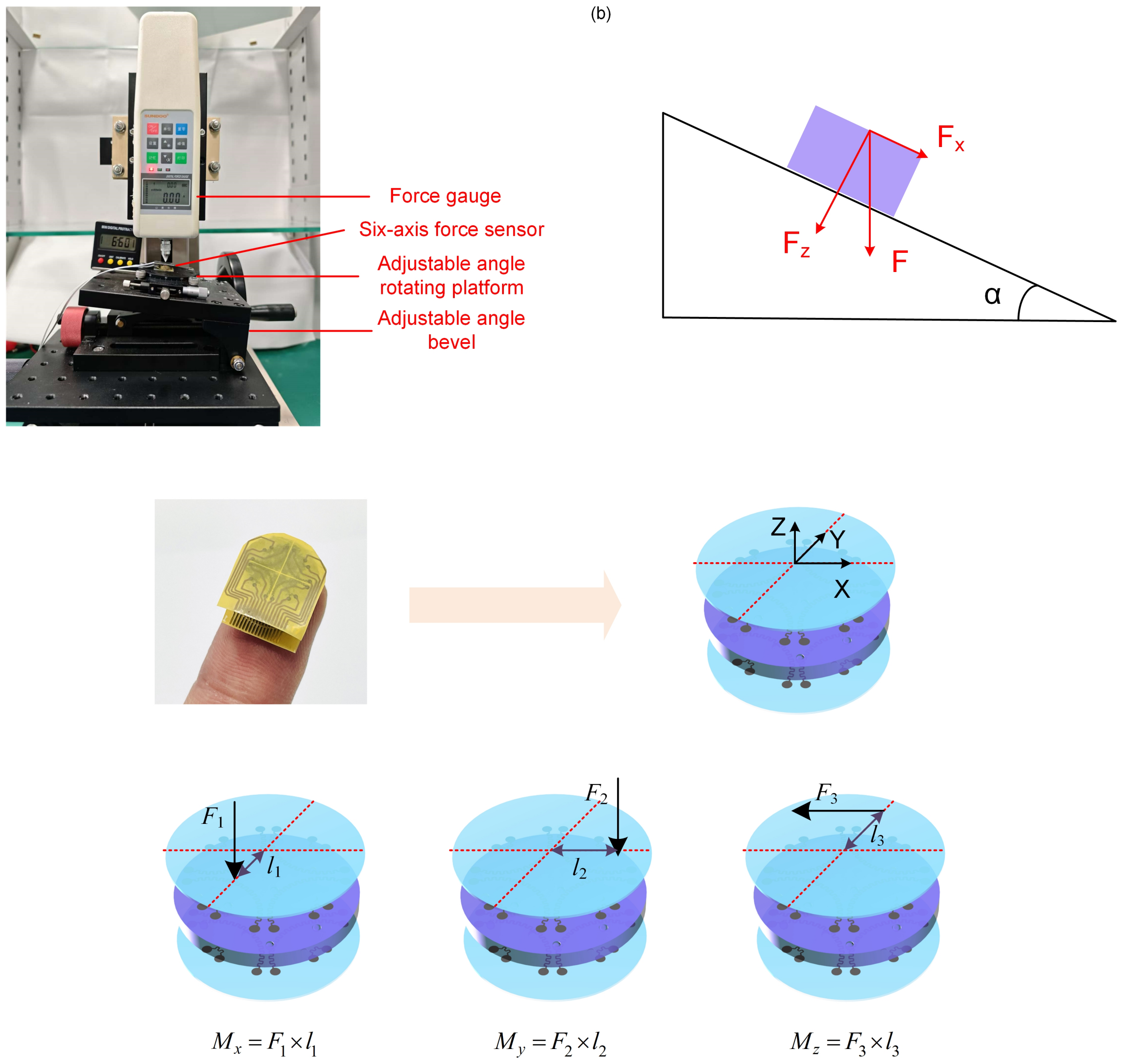

Fig. S6. The testing system for the six-axis force/torque sensor. (a) The photograph of the testing device. (b) The schematic diagram of the testing device. (c) The schematic diagram of the torque calculation.

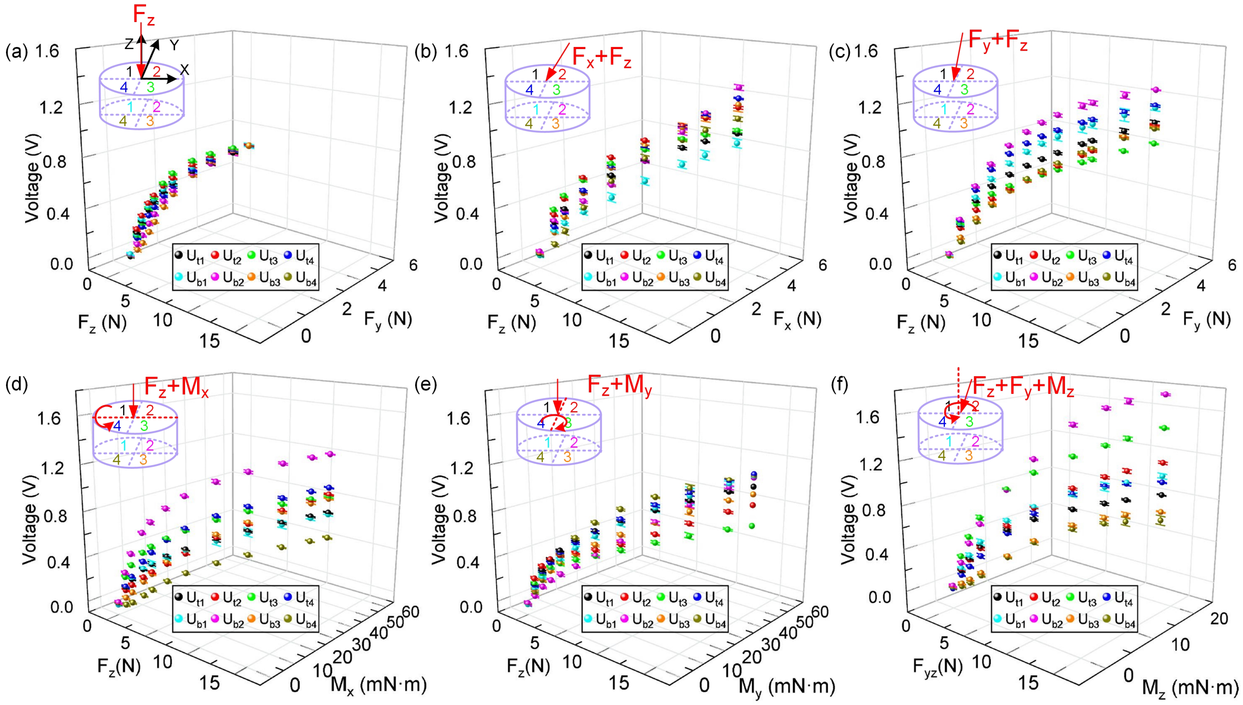

Fig. S7. The responses of the six-axis force/torque sensor under different stimuli. (a) The responses of the six-axis force sensor under F_z. (b) The responses of the six-axis force sensor under F_x and F_z. (c) The responses of the six-axis force sensor under F_y and F_z. (d) The responses of the six-axis force sensor under M_x and F_z. (e) The responses of the six-axis force sensor under M_y and F_z. (f) The responses of the six-axis force sensor under M_z, F_y and F_z. Error bars are standard deviations of three repeated measurements of one specific sensor.

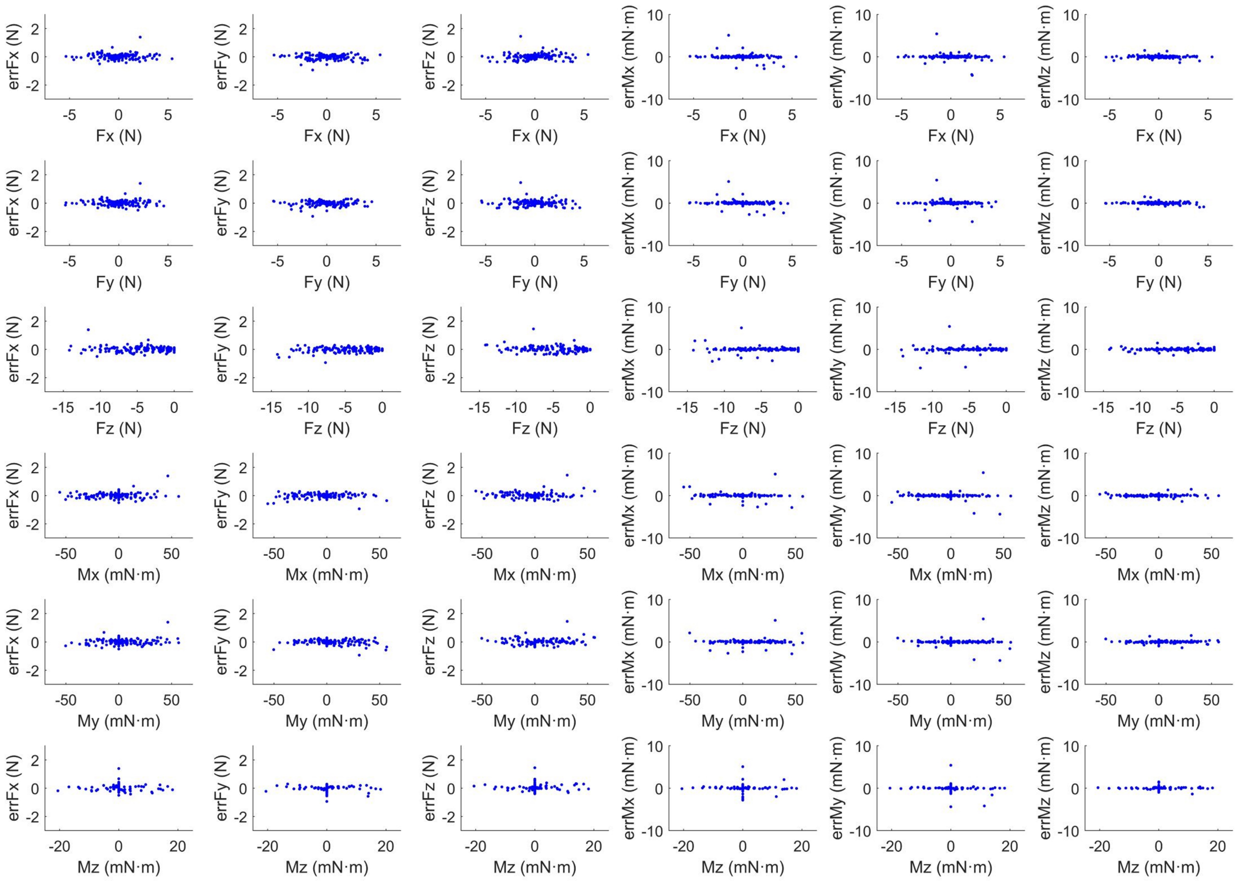

Fig. S8. Cross-talks between different forces and torques. "errFx" refers to the error of F_x, and the others are similar. The results show the error of the sensor when combined loads are applied. For example, the graph in the first row and sixth column shows the measurement error of the torque M_z when the applied force F_x varies from -5 to 5 N. The graph in the third row and the fourth column shows the measurement error of the torque M_x when the applied force F_z varies from -15 to 0 N.

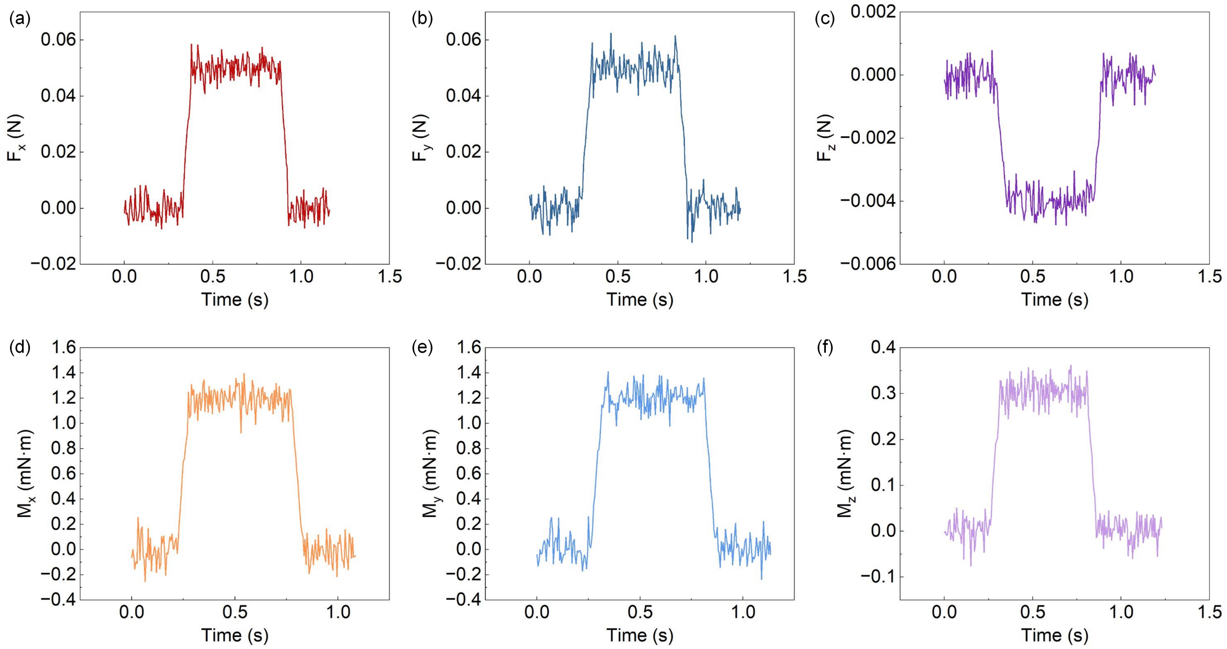

Fig. S9. Detection limits of six-axis force/torque. (a) The detection limit of F_x, while F_z =0.3 N is applied. (b) The detection limit of F_y, while F_z =0.3 N is applied. (c) The detection limit of F_z. (d) The detection limit of M_x, while F_z =0.3 N is applied. (e) The detection limit of M_y, while F_z =0.3 N is applied. (f) The detection limit of F_x, while F_z =0.3 N is applied. The detection limits of F_x, F_y, F_z, M_x, M_y, M_z are 0.052 N, 0.05 N, -0.004 N, 1.20 mN·m, 1.22 mN·m and 0.31 mN·m, respectively.

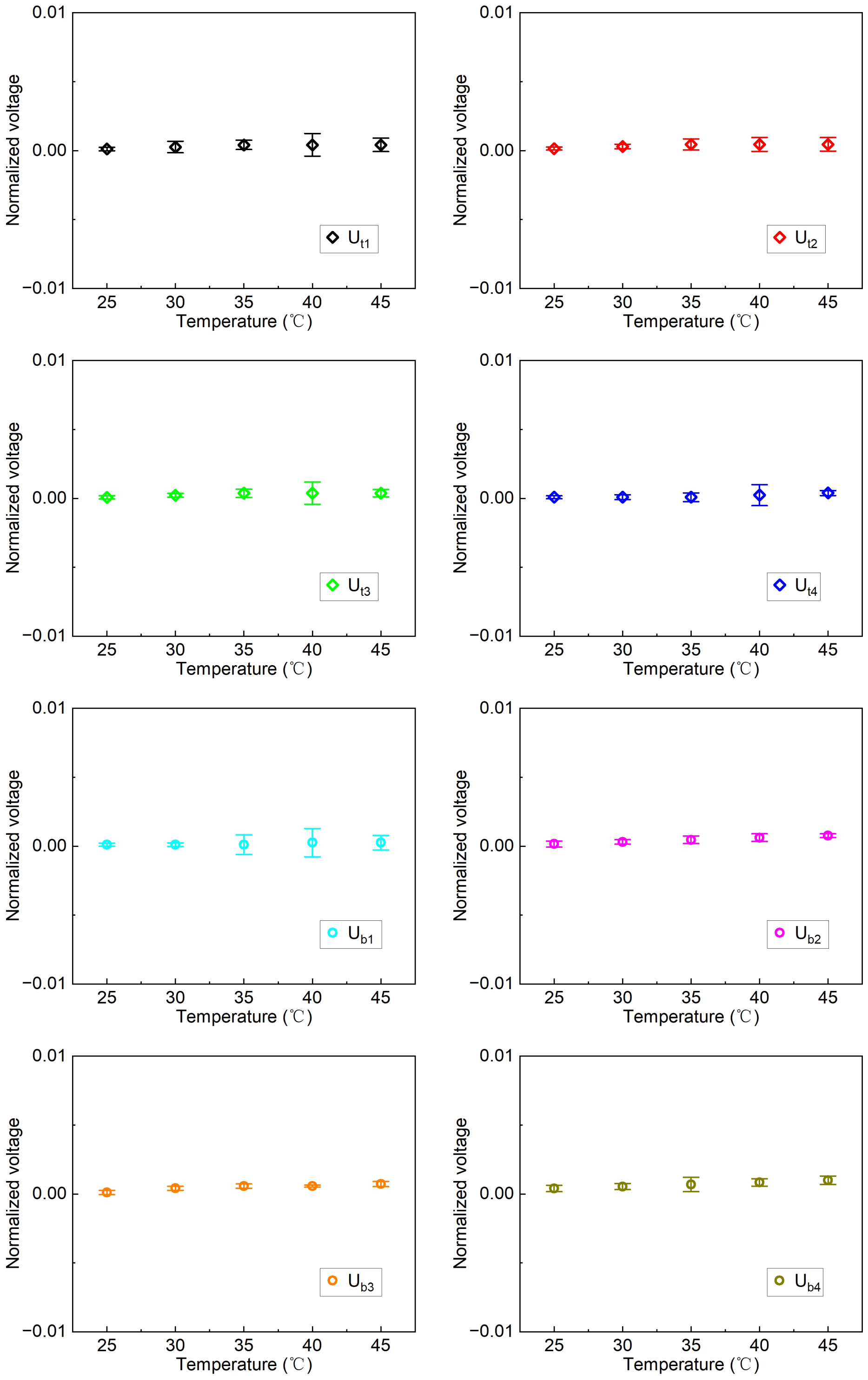

Fig. S10. Sensors' responses under different temperatures varying from 25 ℃ to 45 ℃. Error bars are standard deviations of five repeated measurements of one specific sensor.

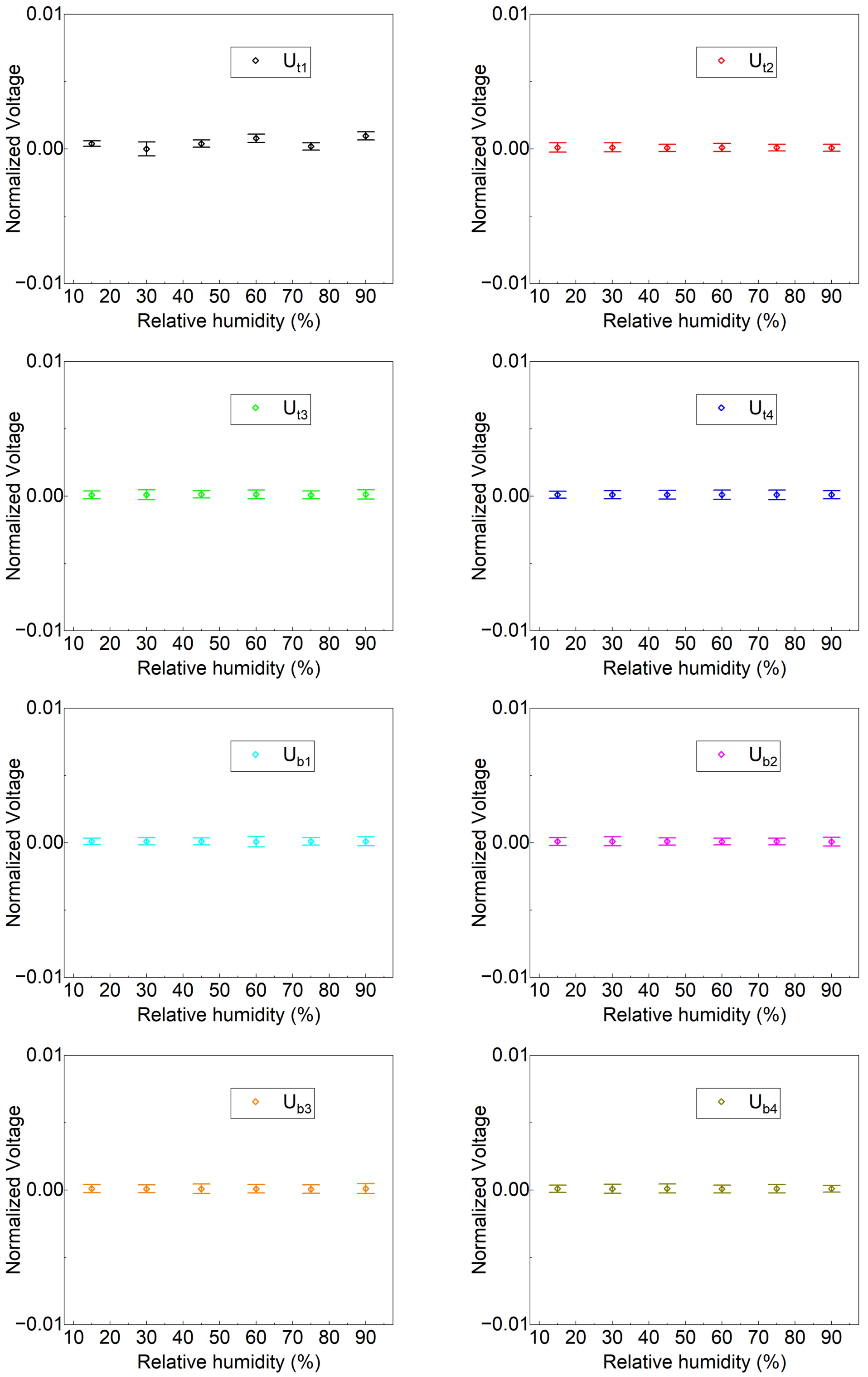

Fig. S11. Sensors' responses under different relative humidity varying from 15% to 90%. Error bars are standard deviations of five repeated measurements of one specific sensor.

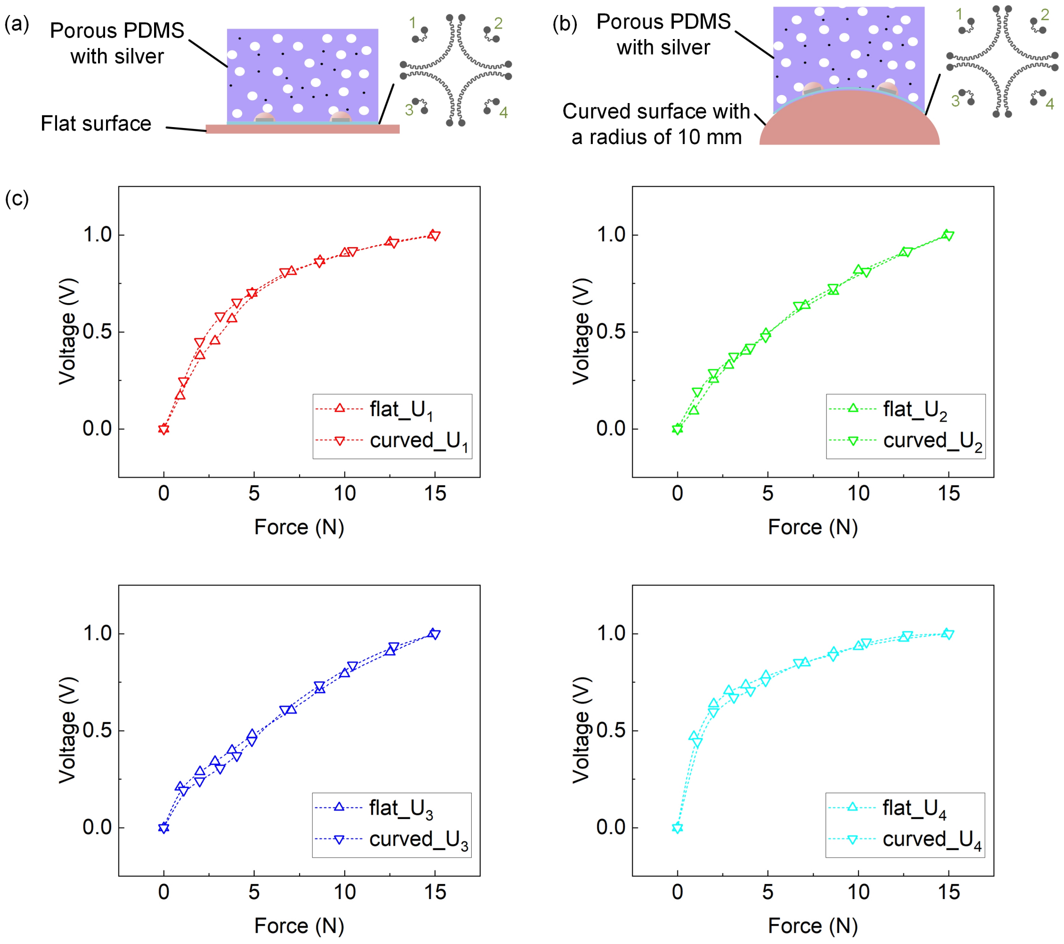

Fig. S12. Sensor test on a flat surface and a curved surface. (a) Schematic diagram of the sensor on a flat surface. (b) Schematic diagram of the sensor on a curved surface with a radius of 10 mm. (c) Sensors' responses comparison on a flat surface and a curved surface. The results indicate that the thermal responses on the flat surface and the curved surface are basically the same, and the impact of bending can be ignored.

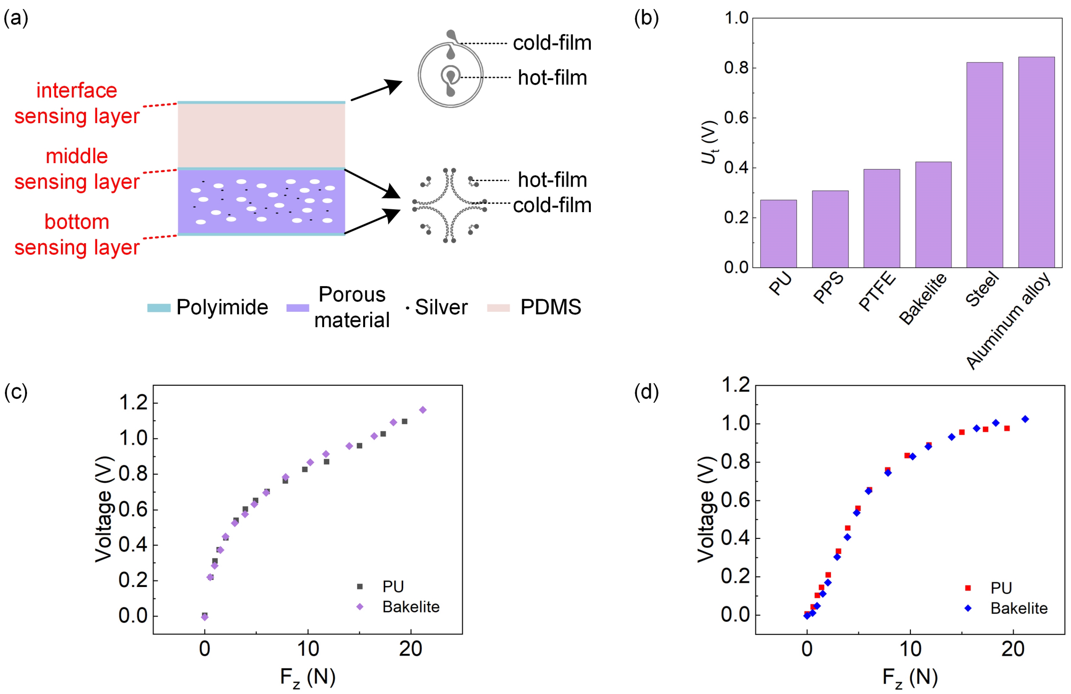

Fig. S13. More perceptive capabilities of force/torque sensor by integrating an interface sensing layer on the top. (a) The structure of the sensor with an interface sensing layer. (b) Responses of the interface sensing layer under material recognition. The interface sensing layer in direct contact with a material detects the thermal attributes of the material (the output signal is denoted as U_t), which provides a means to identify the material type since different material has different thermal attributes. (c) Interference between the interface sensing layer and the middle sensing layer. When the sensor contacts with different materials, the voltage of the middle sensing layer shows no difference, indicating a low interference. (d) Interference between the interface sensing layer and the bottom sensing layer. When the sensor contacts with different materials, the voltage of the bottom sensing layer shows no difference, indicating a low interference. **The results show the low cross-coupling effect of multimodal perceptions.**

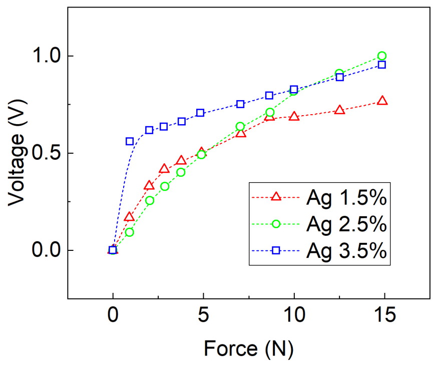

Fig. S14. Optimization of volume ratio of silver nanoparticles. As the volume ratio of silver nanoparticles increases from 1.5% to 3.5%,

Table S1.

Ref.	Flexible	Working principles	Weight	Measuring range	Error
[19]	Yes	Capacitive	/	F_x: ±3.3 N<br>F_y: ±3.3 N<br>F_z: -50~0 N<br>M_z: ±0.015 N·m	F_x: RMSE=0.17 N<br>F_y: RMSE=0.31 N<br>F_z: RMSE=0.20 N<br>M_z: RMSE=0.5 mN·m
[20]	No	Capacitive	2.6 g	F_x: ±30 N<br>F_y: ±30 N<br>F_z: ±30 N<br>M_x: ±0.3 N·m<br>M_y: ±0.3 N·m<br>M_z: ±0.3 N·m	F_x: 0.92%<br>F_y: 0.83%<br>F_z: 1.57%<br>M_x: 1.62%<br>M_y: 1.71%<br>M_z: 0.57%
[21]	No	Capacitive	15g	F_x: ±50 N<br>F_y: ±50 N<br>F_z: ±50 N<br>M_x: ±1 N·m<br>M_y: ±1 N·m<br>M_z: ±1 N·m	F_x: 0.75%<br>F_y: 0.92 %<br>F_z: 1.18%<br>M_x: 1.72%<br>M_y: 1.86%<br>M_z: 0.33%
[29]	Yes	Visual	/	F_x: 0~2 N<br>F_y: 0~2 N<br>F_z: 0~2 N	F_x: 1.5%<br>F_y: 1.5%<br>F_z: 1.5%
[30]	Yes	Visual	/	F_x: ±5 N<br>F_y: ±5 N<br>F_z: -25~0 N<br>M_z: ±0.3 N·m	F_x: 1.5%, RMSE=0.327 N<br>F_y: 1.0%, RMSE=0.309 N<br>F_z: 1.3%, RMSE=1.856 N<br>M_z: 2.3%, RMSE=4.06 mN·m
[31]	Yes	Visual	/	F_x: ±2 N<br>F_y: ±2 N<br>F_z: -6~0 N	F_x: RMSE=0.24 N<br>F_y: RMSE=0.20 N<br>F_z: RMSE=0.32 N
[34]	No	FBG optoelectronic cross-beam	1425 g	F_x: ±10 N<br>F_y: ±10 N<br>F_z: ±25 N<br>M_x: ±1.56 N·m<br>M_y: ±1.56 N·m<br>M_z: ±1.56 N·m	F_x: 1.5%<br>F_y: 1.0%<br>F_z: 1.3%<br>M_x: 1.4%<br>M_y: 1.0%<br>M_z: 2.3%
[41]	No	Strain gauge	/	F_x: ±5 N<br>F_y: ±5 N<br>F_z: ±50 N<br>M_x: ±0.1 N·m<br>M_y: ±0.1 N·m<br>M_z: ±0.1 N·m	F_x: 0.86%<br>F_y: 1.41%<br>F_z: 1.42%<br>M_x: 1.92%<br>M_y: 2.74%<br>M_z: 0.72%
This work	Yes	Thermosensitive	0.30 g	F_x: ±5 N<br>F_y: ±5 N<br>F_z: -15~0 N<br>M_x: ±0.06 N·m<br>M_y: ±0.06 N·m<br>M_z: ±0.02 N·m	F_x: 2.4%, RMSE=0.18 N<br>F_y: 2.02%, RMSE=0.15 N<br>F_z: 0.76%, RMSE=0.18 N<br>M_x: 0.38%, RMSE=0.58 mN·m<br>M_y: 0.35%, RMSE=0.62 mN·m<br>M_z: 0.77%, RMSE=0.26 mN·m

Supplementary Movie 1.
Six-axis force/torque sensor helps robots uncap a bottle.

Supplementary Movie 2.
Six-axis force/torque sensor enables remote control.

Supplementary Movie 3.
Six-axis force/torque sensor enables robotic housekeeping.
