# Supplementary material for: An ultralight, tiny, flexible six-axis force/torque sensor enables dexterous fingertip manipulations
Source: Nat Commun. 2025 Jul 1;16:5693. doi: 10.1038/s41467-025-60861-8 (PMC12214963; doi:10.1038/s41467-025-60861-8)
Supplement: Supplementary file 2 — Description of Additional Supplementary Information [file 41467_2025_60861_MOESM2_ESM.pdf]

**Description of Additional Supplementary Files**

File Name: Supplementary Movie 1
Description: Six-axis force/torque sensor helps robots uncap a bottle.

File Name: Supplementary Movie 2
Description: Six-axis force/torque sensor enables remote control.

File Name: Supplementary Movie 3
Description: Six-axis force/torque sensor enables robotic housekeeping.
